# Inequitable childhood immunization uptake in Nigeria: a multilevel analysis of individual and contextual determinants

**DOI:** 10.1186/1471-2334-9-181

**Published:** 2009-11-20

**Authors:** Diddy Antai

**Affiliations:** 1 Division of Epidemiology, Institute of Environmental Medicine, Karolinska Institute, Stockholm, Sweden; 2 Division of Global Health & Inequalities, The Angels Trust - Nigeria, Abuja, Nigeria

## Abstract

**Background:**

Immunization coverage in many parts of Nigeria is far from optimal, and far from equitable.

Nigeria accounts for half of the deaths from Measles in Africa, the highest prevalence of circulating wild poliovirus in the world, and the country is among the ten countries in the world with vaccine coverage below 50 percent. Studies focusing on community-level determinants therefore have serious policy implications

**Methods:**

Multilevel multivariable regression analysis was used on a nationally-representative sample of women aged 15-49 years from the 2003 Nigeria Demographic and Health Survey. Multilevel regression analysis was performed with children (level 1) nested within mothers (level 2), who were in turn nested within communities (level 3).

**Results:**

Results show that the pattern of full immunization clusters within families and communities, and that socio-economic characteristics are important in explaining the differentials in full immunization among the children in the study. At the individual level, ethnicity, mothers' occupation, and mothers' household wealth were characteristics of the mothers associated with full immunization of the children. At the community level, the proportion of mothers that had hospital delivery was a determinant of full immunization status.

**Conclusion:**

Significant community-level variation remaining after having controlled for child- and mother-level characteristics is indicative of a need for further research on community-levels factors, which would enable extensive tailoring of community-level interventions aimed at improving full immunization and other child health outcomes.

## Background

Child mortality has fallen significantly in many low-income countries; however, sub-Saharan Africa continues to experience the slowest fall in mortality rate among children [1]. It is estimated that 10.8 million child die worldwide each year, of which 41% of these deaths occur in sub-Saharan Africa and 34% in south Asia [2]. Six countries - India, Nigeria, China, Pakistan, the Democratic Republic of the Congo and Ethiopia - account for half of worldwide childhood deaths, Nigeria is ranked 2^nd ^overall, and 17^th ^when ranked by under-five mortality rate [3]. The millennium development goals (MDG) for health, in 2002, set targets for nations to reduce under-five mortality rate by two-thirds by 2015, from the base year 1990 [4]. According to Nigeria's first Millennium Development Goals Report in December 2004, Nigeria has already missed the 2005 target of the Goal for gender. The country may however not meet the other goals by 2015, unless current trends are reversed [5]. Among the reasons for slow progress in attaining the goal for reduction in child mortality in Nigeria are the inequitable access to immunization services, deficient vaccine supplies and equipments [6]. Current coverage rates for the various childhood vaccines in Nigeria are among the lowest in the world [7]. For instance, Measles was responsible for 5 percent of the child deaths in Africa [8], of an estimated 282 000 deaths in 2003 [9,10]; half of these occurred in Nigeria [11]. Nigeria is among the ten countries in the world with vaccine coverage rates below 50 percent [11], having been persistently below 40 percent since 1997 [10]. The country also has the highest prevalence of circulating wild poliovirus in the world [10,12].

Vaccines are among the most effective preventive health measures in reducing child mortality, morbidity, and disability [13,14]. The introduction of appropriate vaccines for routine use on infants has resulted in drastic reductions in vaccine-preventable diseases [3,15]. The Expanded Program on immunization (EPI) in middle- and low-income countries has prevented more than 2 million child deaths from the Tuberculosis, Diphtheria, Tetanus, Pertussis, Polio, and Measles each year since its initiation in 1974 [16]. With the establishment of the Global Polio Eradication Initiative in 1988, immunization has resulted in a 99 percent reduction in the worldwide incidence of poliomyelitis [16,17]. By reducing morbidity and mortality, Immunization is expected to contribute significantly to the achievement of the Millennium Development Goal 4 (to achieve a tow-thirds reduction in mortality rates for children under the age of 5 years between 1990 and 2015 [18].

Nigeria's routine immunization schedule stipulates that infants should be vaccinated with the following vaccines: a dose of Bacillus Calmette-Guerin (BCG) vaccine at birth (or as soon as possible); three doses of Diphtheria, Pertussis and Tetanus (DPT) vaccine at 6, 10 and 14 weeks of age; at least three doses of oral Polio vaccine (OPV) - at birth, and at 6, 10 and 14 weeks of age; and one dose of Measles vaccine at 9 months of age (Table 1). The country's immunization programmes have however been characterized by intermittent failures and successes since the initial introduction in 1956. Immunization programmes were again re-introduced as the Expanded Programme on Immunization (EPI) in 1979 to provide immunization services to children aged 23 months and younger. Following repeated and limited initial success, the immunization programme was re-launched in 1984. Studies show that individual, community and systemic factors affect the equitable uptake of childhood immunization in Nigeria, as in other countries in sub-Saharan Africa [19]. Widespread inequities persist in immunization coverage to the disadvantage of children of parents in the lowest socio-economic quintile, parents with no education, and parents residing in rural areas, especially in the Northern regions [20]. Inequitable access to routine immunization in Nigeria has also been attributed to fear and confusion [7]. It is on the background of these mitigating factors that this study aims to: (1) assess the individual-level determinants of full immunization, by sequentially controlling for explanatory factors; and (2) determine whether community-level explanatory factors account for variations in full immunization.

**Table 1:** Routine expanded programme on immunization (EPI) schedule in Nigeria

Vaccine	Schedule
Bacillus Calmette-Guerin (BCG)	Birth
Diphtheria, pertussis and tetanus (DPT) I	6 weeks
Oral polio vaccine (OPV) I	6 weeks
Diphtheria, pertussis and tetanus (DPT) II	10 weeks
Oral polio vaccine (OPV) II	10 weeks
Diphtheria, pertussis and tetanus (DPT) III	14 weeks
Oral polio vaccine (OPV) III	14 weeks
Measles	9 months

## Methods

Data on the health and mortality of children in Nigeria were collected as part of the Nigeria Demographic and Health Survey (DHS). This study uses data from the 2003 edition of this survey, which is a nationally-representative probability sample, collected using a stratified two-stage cluster sampling procedure. Sampling of women was performed according to the list of enumeration areas developed from the 1991 Population Census sampling frame. The initial sampling stage involved selecting 365 clusters, also known as primary sampling units (PSUs) with a probability proportional to the size. The size, in this case, is the number of households in the cluster. Subsequent sampling involved systematically selecting households from the already selected clusters. This resulted in a probability sample of 7864 households, from which data was collected by face-to-face interviews from 3725 women aged 15 to 49 years. These women contributed a total of 6029 live born children born to the survey. Information collected included birth histories, in-depth demographic and socio-economic information on illnesses, medical care, immunizations, and anthropometric details of children [20]. Immunization status of a child was determined from vaccination cards shown to the DHS interviewer. In the absence of vaccination cards, mothers were asked to recall whether the child had received BCG, Polio, DPT (including the number of doses for each) and Measles vaccinations.

### Measures

#### Outcome

The outcome variable is the likelihood of a child 12 months of age and older having received all of the eight required vaccinations (full immunization).

### Exposures

#### Individual-level risk factors

Eight additional child- and mother-level variables of interest were examined: *i) *sex of the child, assessed as: male and female; *ii) *birth order and interval between births, created by merging "birth order" and "preceding birth interval" classified as: first births, birth order 2-4 with short birth interval (<24 months), birth order 2-4 with medium birth interval (24-47 months), birth order 2-4 with long birth interval (48+ months), birth order 5+ with short birth interval (<24 months), birth order 5+ with medium birth interval (24-47 months), and birth order 5+ with long birth interval (48 months); *iii) *mothers' age, grouped as: 15-18, 19-23, 24-28, 29-33, and 34 years and older; *iv) *marital status, grouped as: single, married, and divorced; *iv) *ethnicity, categorized as: a) Hausa/Fulani/Kanuri (grouped on the basis that these ethnic groups either speak a common language or dialect, share a common sense of identity, cohesion and history; or have a single set of customs and behavioural rules as in marriage, clothing, diet, taboos); b) Igbo; c) Yoruba; and d) Others (a merger of various other minority ethnic groups from the more than 374 identifiable ethnic groups in Nigeria); *v) vi) *mothers' education, categorized as: no education, primary, and secondary or higher education; *vii) *mothers' occupation, categorized as: professional/technical/managerial, clerical/sales/services/skilled manual, agricultural self-employed/agricultural employee/household & domestic/unskilled manual occupations, and not working; and *viii) *mothers' household wealth index, categorized into five quintiles as: poorest, poorer, middle, richer and richest.

#### Community-level risk factors

Primary sampling units or clusters are administratively-defined areas used as proxies for "neighbourhoods" or "communities" [21,22], and are relevant when the hypothesis involves policies. Primary sampling units are small and designed to be fairly homogenous units with respect to population socio-demographic characteristics, economic status and living conditions, and consist of one or more enumeration areas (EAs), which are the smallest geographic units for which census data are available in Nigeria. Each cluster was made up of a minimum of 50 households; in the case of less than 50 households, a contiguous enumeration area was added [20]. Four community-level variables were assessed. Community prenatal care by doctor was assessed because prenatal care directly increases the chances that mothers would access subsequent health care services for their child, such as institutional delivery and immunization [23,24]. Community hospital delivery was included because the proportion of mothers that delivered in a hospital setting is a predictor of child immunization uptake. Hospital delivery is one of the most important preventive measures against maternal and child health outcomes, and an important determinant of full immunization [25,26]. Community mother's education was assessed because higher levels of maternal education are associated with better child health outcomes, such as child immunization rates [23,24]. These community-level variables were: *i) *community mother's education, defined as the percentage of mothers with secondary or higher education in the primary sampling unit, and categorized as: low, middle, and high (cut-off at median value in all primary sampling units combined; "middle" referring to the proportion at the median value, "low" referring to the proportion below the median value, and "high" referring to the proportion above the median value); *ii) *community hospital delivery, defined as the percentage of mothers who delivered their child in the hospital, and categorized as: low, middle, and high (cut-off at median value in all primary sampling units combined); *iii) *Community prenatal care by doctor, defined as the percentage of mothers who received prenatal care by a doctor and categorized as: low, and high (cut-off at 13% in all primary sampling units combined); and *iv) *mother's region of residence, categorized according to the six geo-political zones in Nigeria, as: North Central, North East, North West, South East, South South, and South West. Community-level variables were estimated at the level of the primary sampling unit (*n *= 365).

### Statistical analysis

The distribution of the children and mothers in the sample by full immunization status was assessed. Normalized sample weights provided in the DHS data were used for all analyses using Stata 10 software package [27], so as to adjust for non-response and enable generalization of findings to the general population. A three-level multilevel logistic regression model was applied in order to account for the hierarchical structure of the DHS data [28]. Children (level 1), were nested within mothers (level 2), who were in turn nested within communities (level 3).

Four models containing variables of interest were fitted. **Model 0 **(empty model) contained no exposure variable and only focused on decomposing the total variance into its mother and community components. **Model 1 **contained child-level variables (sex of the child, birth order/birth interval of the children) and **Model 2 **included mother-level variables (mothers' age, marital status, ethnicity, mothers' education, mothers' occupation, and mothers' household wealth index). **Model 3 **contained community-level variables (community mother's education, community hospital delivery, community prenatal care by doctor, and mothers' region of residence).

The three-level multilevel model is written as follows:(1)

where ***π***_*ijk *_is the probability of dying for the *i*th child of the *j*th mother in the *k*th community, ***e***_*ijk *_is a child-level error term distributed as Bernoulli constant, ***X***_*ijk *_is a vector of covariates corresponding to the *i*th child of the *j*th mother in the *k*th community including mother's ethnicity, and educational background, ***β***_0 _is a vector of unknown parameters, ***u***_0jk _is the random effect at the mother level, and ***v***_0k _is the random effect at the community level.

The intercept or average probability of being fully immunized is assumed to vary randomly across mothers and communities. The fixed effects (measures of association) are expressed as odds ratio (OR) and 95% confidence intervals (95% CI). The random effects (measures of variation) are expressed as Variance Partition Coefficient (VPC) and proportional change in variance (PCV). We appraised the precision by the standard error (SE) of the explanatory variables, and tested parameters using the Wald statistic i.e. the ratio of the estimated variance to its standard error [29], and we calculated *p*-values. MLwiN software package 2.0.2 [30] was used for the multilevel analyses, with Binomial, Penalized Quasi-Likelihood (PQL) procedures [31]. Missing data were excluded from the analysis.

### Ethical considerations

This study is based on analysis of secondary data with all participant identifiers removed. The survey was approved by the National Ethics Committee in the Federal Ministry of Health, Nigeria and the Ethics Committee of the Opinion Research Corporation Macro International, Incorporated (ORC Macro Inc.), Calverton, USA. Informed consent was obtained from the participants prior to participation in the survey, and data collection was done confidentially. Permission to use the DHS data in this study was obtained from ORC Macro Inc.

## Results

### Proportion of children that received full immunization by individual-level characteristics (Table 2)

**Table 2:** Proportion of children that received full immunization

Individual characteristics	Full immunization		
	
	Yes	No	
	
	*n *(%)	*n *(%)	Total *N *(%)
*Sex of child*			
Female	249 (13)	1617 (87)	1866 (100)
Male	256 (14)	1609 (86)	1865 (100)
*Birth order/birth interval*			
First birth (order 1)	108 (14)	638 (86)	746 (100)
Order 2-4 & <24 months	59 (14)	359 (86)	418 (100)
Order 2-4 & 24-47 months	146 (15)	848 (85)	994 (100)
Order 2-4 & 48+ months	32 (14)	204 (86)	236 (100)
Order 5+ & < 24 months	22 (8)	239 (92)	261 (100)
Order 5+ & 24-47 months	99 (12)	695 (88)	794 (100)
Order 5+ & 48+ months	39 (14)	245 (86)	284 (100)
*Mother's age*			
15-18	10 (5)	200 (95)	210 (100)
19-23	91 (11)	699 (89)	790 (100)
24-28	154 (14)	973 (86)	1127 (100)
29-33	102 (14)	637 (86)	739 (100)
≥ 34	148 (17)	717 (83)	865 (100)
*Marital status*			
Single	13 (21)	49 (79)	62 (100)
Currently married	473 (13)	3076 (87)	3549 (100)
Formerly married	19 (16)	101 (84)	120 (100)
*Ethnicity*			
Hausa/Fulani/Kanuri	128 (7)	1774 (93)	1902 (100)
Igbo	84 (23)	285 (77)	369 (100)
Yoruba	84 (34)	165 (66)	249 (100)
Others	209 (17)	1002 (83)	1211 (100)
*Mothers' education*			
No education	169 (8)	1986 (92)	2155 (100)
Primary	142 (18)	663 (82)	805 (100)
Secondary or higher	194 (25)	577 (75)	771 (100)
*Mothers' occupation*			
Professional/Technical/Management	41 (36)	74 (64)	115 (100)
Clerical, sales, services, skilled manual	218 (14)	1385 (86)	1603 (100)
Agric. self., Agric. employee, household & domestic, unskilled manual	97 (17)	483 (83)	580 (100)
Not working	149 (10)	1284 (90)	1433 (100)
*Wealth index*			
Poorest	82 (9)	869 (91)	951 (100)
Poorer	79 (9)	765 (91)	844 (100)
Middle	96 (12)	681 (88)	777 (100)
Richer	98 (15)	574 (85)	672 (100)
Richest	150 (31)	337 (69)	487 (100)

The uptake of full immunization among the children was generally low. Children of high birth order 5+ with short birth order (<24 months), children of younger mothers, and Hausa/Fulani/

Kanuri mothers had remarkably lower (less than 10%) likelihood of receiving full immunization. Children of mothers with lower socio-economic status (mothers with no education, who were not working, and in the poorest wealth quintile) had lower likelihood of receiving full immunization.

### Multilevel logistic regression analysis (Table 3)

**Table 3:** Odds ratios and 95% confidence intervals for multilevel logistic regression models

Variables	Model 0 (Empty model)	Model 1 (Child-level variables)	Model 2 (Mother-level variables)	Model 3 (Community-level variables)
	**OR (95% CI)**	**OR (95% CI)**	**OR (95% CI)**	**OR (95% CI)**

**Fixed effects**				
***Individual characteristics***				
*Sex of child*				
Female		1.02 (0.85-1.22)	1.01 (0.83-1.22)	1.00 (0.80-1.26)
Male		1	1	1
*Birth order/birth interval*				
First birth (order 1)		1.03 (0.79-1.34)	0.98 (0.72-1.33)	0.99 (0.69-1.41)
Order 2-4 & <24 months		1.00 (0.73-1.38)	1.03 (0.73-1.44)	0.89 (0.62-1.31)
Order 2-4 & 24-47 months		1	1	1
Order 2-4 & 48+ months		0.78 (0.52-1.17)	0.66 (0.43-1.01)	0.65 (0.39-1.07)
Order 5+ & < 24 months		0.51 (0.33-0.79)*	0.54 (0.33-0.89)	0.61 (0.34-1.09)
Order 5+ & 24-47 months		0.85 (0.65-1.11)	0.84 (0.60-1.18)	0.87 (0.59-1.30)
Order 5+ & 48+ months		0.98 (0.67-1.42)	0.84 (0.54-1.32)	0.70 (0.41-1.21)
*Mother's age*				
15-18			0.55 (0.27-1.11)	0.45 (0.19-1.10)
19-23			0.99 (0.73-1.36)	0.89 (0.62-1.29)
24-28			1	1
29-33			0.98 (0.72-1.32)	0.81 (0.57-1.16)
≥ 34			1.33 (0.95-1.85)	1.33 (0.91-1.94)
*Marital status*				
Single			1.18 (0.52-2.66)	0.92 (0.35-2.40)
Currently married			0.89 (0.53-1.49)	0.67 (0.37-1.19)
Formerly married			1	1
*Ethnicity*				
Hausa/Fulani/Kanuri			1	1
Igbo			1.66 (1.15-2.41)	2.47 (1.28-4.76)
Yoruba			1.90 (1.29-2.81)	1.47 (0.78-2.80)
Others			1.68 (1.27-2.23)	1.47 (0.96-2.25)
*Mothers' education*				
No education			0.85 (0.62-1.16)	0.85 (0.58-1.25)
Primary			1.05 (0.79-1.39)	1.04 (0.74-1.48)
Secondary or higher			1	1
*Mothers' occupation*				
Prof./Tech./Manag.			1	1
Clerical, sales, services, skilled manual			0.62 (0.40-0.96)	0.56 (0.34-0.93)
Agric. self., Agric. employee, household & domestic, unskilled manual			0.81 (0.49-1.33)	0.88 (0.49-1.60)
Not working			0.68 (0.43-1.08)	0.68 (0.40-1.16)
*Wealth index*				
Poorest			0.44 (0.29-0.65)	0.35 (0.21-0.59)
Poorer			0.49 (0.34-0.71)	0.47 (0.27-0.81)
Middle			0.66 (0.47-0.93)	0.64 (0.42-0.97)
Richer			0.63 (0.46-0.87)	0.57 (0.39-0.84)
Richest			1	1
***Community characteristics***				
*Community mother's education*				
Low				0.76 (0.55-1.06)
Middle				1
High				1.02 (0.73-1.41)
*Community hospital delivery*				
Low				0.62 (0.40-0.94)
Middle				1
High				1.12 (0.75-1.68)
*Community prenatal care by doctor*				
Low				1.20 (0.79-1.84)
High				1
*Region of residence*				
North Central				1.15 (0.69-1.91)
North East				0.90 (0.48-1.70)
North West				0.83 (0.42-1.67)
South East				0.36 (0.18-0.73)
South South				0.48 (0.26-0.87)
South West				1
**Random effects**	*Empty*	*Child-level*	*Mother-level*	*Community-level*
*Community-level*				
Variance (SE)	0.393 (0.119)**	0.567 (0.189)**	0.210 (0.112)	0.272 (0.133)*
VPC (%)	9.7	13.6	4.9	13.9
Explained variation (PVC) (%)	Reference	44.3	63	-29.5
*Mother-level*				
Variance (SE)	0.382 (0.193)*	0.296 (0.145)*	0.790 (0.216)*	0.576 (0.241)*
VPC (%)	9.4	7.1	18.4	13.9
Explained variation (PVC) (%)	Reference	22.5	-170	27
**Model fit statistics**				
DIC	3341	3337	3158	2269

Note: Model 0 contained no variables; Model 1 included child-level characteristics; Model 2 adjusted for mother-level characteristics; Model 3 additionally adjusted for community-level characteristics.

Abbreviations: VPC = Variance partition coefficient; DIC = Deviance information criterion; SE = Standard error: OR = Odds ratio; CI = Confidence Interval.

Data source: 2003 Nigeria Demographic and Health Survey

*p < .05; **p < .01; ***p < .001

The total variance in full immunization associated with contexts was initially estimated using the empty model, which contains no variables and only partitions the total variance in full immunization into the sum of the individual-level and contextual-level variances, and as such provides an estimate of intra-class correlation coefficient or variance partition coefficient. The variance was significant across mothers (τ = 0.382, *p *= 0.048) and communities (τ = 0.393, *p *= 0.001). As indicated by the variance partition coefficient, the intra-mother and intra-community correlations are 9.4% and 9.7% respectively.

Sex of the child and birth order/birth interval were introduced in Model 1 as the child-level covariates, and their slopes were allowed to vary in order to investigate whether their effects are different across contexts. Children of birth order 5+ with short birth interval (<24 months) had a 49% lower likelihood of receiving full immunization (OR = 0.51, 95% CI = 0.33 - 0.79) compared with the reference group. In comparison to the empty model, the variation in full immunization in Model 1 remained significant across mothers (τ = 0.296, *p *= 0.041) and communities (τ = 0.567, *p *= 0.003). The intra-mother correlation was 7.1% and the intra-community correlation was 13.6%. The proportional change in variance of the odds of full immunization of 22.5% across mothers and 44.3% across communities was explained by child-level compositional factors, and indicates that part of the clustering of full immunization within areas is due to composition of the communities by birth order/interval of the children.

With the introduction of mothers' age, marital status, ethnicity, mothers' education, mothers' occupation and mothers' household wealth index in Model 2, the likelihood of being fully immunized was higher for children of mothers from the Igbo (OR = 1.66, 95% CI = 1.15 - 2.41), Yoruba (OR = 1.90, 95% CI = 1.29 - 2.81), and Other (OR = 1.68, 95% CI = 1.27 - 2.23) ethnic groups compared to children of Hausa/Fulani/Kanuri mothers. Children of mothers working as clerical, sales, services, skilled manual employees had lower likelihood of being fully immunized (OR = o.62, 95% CI = 0.40 - 0.96) compared to children of professional/technical/management employees. The likelihood of a child being fully immunized was lower for children of mothers in the poorest (OR = 0.44, 95% CI = 0.29 - 0.65), poorer (OR = 0.49, 95% CI = 0.34 - 0.71), middle (OR = 0.66, 95% CI = 0.47 - 0.93), and richer (OR = 0.63, 95% CI = 0.46 - 0.87) wealth quintiles compared to children of mothers in the richest wealth quintile.

In comparison to Model 1, the variation in full immunization in Model 2 also remained significant across mothers (τ = 0.790, *p *= 0.012) only. The intra-mother correlation decreased to 18.4% while the intra-community correlation decreased to 4.9%. The proportional change in variance of odds of full immunization of -170% across mothers and 63% across communities was explained by mother-level characteristics, indicating that part of the clustering of full immunization within areas is attributable to the composition of the communities by mother's characteristics.

Finally, Model 3 included community mothers' education, community hospital delivery, community prenatal care by doctor and region of residence. Children of mothers living in communities with low level of hospital delivery had a 38% lower likelihood of receiving full immunization (OR = 0.62, 95% CI = 0.40 - 0.94) compared to children of mothers living in communities with hospital delivery at the median level. Children of mothers living in the South East and South South regions had 64% (OR = 0.36, 95% CI = 0.18 - 0.73) and 52% (OR = 0.48, 95% CI = 0.26 - 0.87) lower likelihood respectively of receiving full immunization than children of mothers living in the South West region.

In comparison to Model 2, the community-level (τ = 0.272, *p *= 0.041) and mother-level variation remained significant (τ = 0.576, *p *= 0.017). Both the variance partition coefficient across mothers and communities increased to 13.9%. The proportional change in variance of the odds of full immunization of 27% across mothers and -29.5% across communities was explained by mother-level characteristics, indicating that community differences in the likelihood of full immunization are partly due to composition of the communities by community-level characteristics. Successively smaller values of Deviance Information Criterion (DIC) with each subsequent model in the bottom of Table 2 show that each model represents a significant improvement over the previous model and indicates the goodness-of-fit of the model used in this analysis.

## Discussion

This study shows that the pattern of full immunization clusters within families as well as within communities. Having taken into consideration the fact that children of the same mother or living within the same community will experience similar likelihood of immunization, the results of this study indicate that individual-level (ethnicity, mothers' occupation, mothers' household wealth) and community-level (proportion of mothers that had hospital delivery) socio-economic characteristics are important in explaining the differences in full immunization among the children in the study. Children of mothers' from the Igbo ethnic group had more than twice the likelihood of receiving full immunization compared to children of Hausa/Fulani/Kanuri mothers. Ethnic differences in Nigeria not only generally reflect differences in social identity, attitudes and health-seeking behaviour, but also reflect disparities in socio-economic position. The Igbos (or Ibos) have high economic power, which is a characteristic that increases their propensity to migrate from areas with poor economic opportunities into areas with higher economic opportunities, more than most other ethnic groups in Nigeria [32]. Increased socio-economic position increases the likelihood of children being fully immunized.

The six geopolitical regions in Nigeria represent different religious and political situations, economic potentials, population densities and levels of development [33]. These regional disparities tend to reflect the range of child immunization campaign effectiveness across the country [34] and across communities, which could be linked with variations in vaccine supply between communities within the different regions. The South East and South South regions in Nigeria are economically deprived regions. The South South (or Niger Delta) region in particular is characterized by extensive mangrove forests, lagoons and swamps stretching over hundreds of kilometres inland, as well as poverty, poor social infrastructure and conflicts that are exacerbated by environmental degradation from crude oil pollution. Many of the children targeted in the vaccination campaigns in the South South region generally reside in impoverished and hard-to-reach settlements across the Niger Delta Region. In addition, vaccination teams face threats from armed militias that roam the area in search of opportunities to seize control over the local oil resources [35]. These conditions make children in these regions inaccessible to vaccination officers, and it is therefore not surprising that this study found that children in the South East and South South regions in Nigeria had significantly lower risks of receiving full immunization.

Socio-economic position (especially education) of individuals and populations strongly influences the behaviour of individuals and thereby influences health-seeking behaviour and ultimately child survival. Higher socio-economic status is associated with better health [36], and this is shown to be true in this study. Though maternal education was not significantly associated with the likelihood of full immunization, household wealth and mothers' occupation are factors that influence vaccination uptake, given that they influence parents' likelihood to seek immunization for their child. This study showed that mothers' occupation (clerical, sales, services, skilled manual) was significantly associated with the lower likelihood of full immunization. This is not surprising, given that people with such occupations are of lower socio-economic status, and need to get permission to take time off work to get their children vaccinated or to seek medical care; this in itself has negative consequences on the risks of full immunization. Mothers' household wealth was significantly and proportionally associated with the likelihood of full immunization, with higher position in the wealth index being associated with increased likelihood of full immunization. Similar findings have been reported in previous studies [34].

This study showed that living in a community with low proportion of mothers with hospital delivery was associated with lower likelihood of full immunization. This association is in line with expectations, given that timely access to maternal healthcare (hospital delivery) is one of the most important preventive measures against maternal and child health outcomes [37,38]. Community hospital delivery is also an indication of the quality of care received by the mother and infant during delivery, and associated with the higher likelihood of full immunization. This is an example of socio-economic position at the community level affecting health outcomes and mimics the associations seen at the individual level. The percentage of hospital deliveries in the community is also an indication of the access to maternal and child health services in general. It is therefore expected that communities with less hospital deliveries also have lower immunization status in general.

Results in this study reflect the need for equity in the focus of immunization programs, with increased involvement of local communities in the conceptualization and implementation of such vaccination programs. In addition, community-based initiatives focusing on the proportion of mothers that receive maternal healthcare services (prenatal and delivery) within communities as well as increasing the proportion of mothers with higher education within communities should be targeted. Community-level variation in the likelihood of full immunization remained significant after controlling for child- and mother-level variables, indicating a need for further exploration of community-levels effects on child immunization uptake.

Findings in this study should however be considered in light of the following limitations. First, the administratively defined boundaries used as a proxy for neighbourhoods in this study may non-differentially misclassify individuals into an inappropriate administrative boundary, which can generate information biases and reduce the validity of analyses. Second, other individual and community factors not addressed in the present study are also likely to be important determinants of full immunization. Third, demographic and health surveys do not ordinarily collect data on household income or expenditure, which are the indicators commonly used to measure wealth. The assets-based wealth index used here is only a proxy indicator for household economic status, which may not always produce results similar to those obtained from direct measurements of income and expenditure where such data are available or can be collected reliably [39].

## Conclusion

The results of this study suggest the need to close individual- and community-level disparities in the likelihood of full immunization, with particular emphasis being placed on interventions needed to increase maternal education, improve maternal knowledge, attitudes and uptake of vaccinations, as well as increase the proportion of mothers receiving prenatal care and hospital delivery. These interventions are expected to aid in increasing childhood vaccination coverage. The relevance of the findings lie in that importance of identifying vulnerable groups with low immunization uptake, as well as the behavioural processes associated with low immunization uptake, so as to enable the implementation of appropriate interventions aimed to increasing full immunization uptake among the culturally diverse and geographically dispersed Nigerian population. Identifying areas or groups that are disadvantaged in immunization services are a prerequisite for the efficient allocation public health resources, and are important for the success of immunization campaigns.

## Competing interests

The author declares that they have no competing interests.

## Pre-publication history

The pre-publication history for this paper can be accessed here:

http://www.biomedcentral.com/1471-2334/9/181/prepub

## References

[B1] Ahmad O Lopez A Ionue O The decline in child mortality: a reappraisal Bull World Health Organ 2000 78 1175 1191 11100613 PMC2560617

[B2] Jones G Steketee RW Black RE Bhutta Z Morris SS the Bellagio Child Survival Study Group How many child deaths can we prevent this year? Lancet 2003 362 62 71 10.1016/S0140-6736(03)13810-X 12853204

[B3] Black RE Morris SS Bryce J Where and why are 10 million children dying every year? Lancet 2003 361 2226 2234 10.1016/S0140-6736(03)13779-8 12842379

[B4] United Nations General assembly, 56^th ^session. Road map towards the implementation of the United Nations millennium declaration: report of the Secretary-General (UN document no. A/56/326) 2001 New York: United Nations

[B5] Alaba OA Alaba OB Malaria in rural Nigeria: Implications for the Millennium Development Goals Africa Development Review 2009 21 73 85 10.1111/j.1467-8268.2009.00204.x

[B6] Lambo E Achieving the Health Millennium Development Goals in Sub-Saharan Africa: The Role of Science and Technology A presentation given at An Africa-Canada-UK Exploration January 30 to February 1, 2005 Canada House, London 2005 http://www.scidev.net/africancapacity/presentations/session5/Lambo5.ppt

[B7] Babalola S Aina O Community and systemic factors affecting the uptake of immunisation in Nigeria: A qualitative study in five states 2004 National Report, Abuja: PATHS

[B8] Bryce J Boschi-Pinto C Shibuya K Black RE WHO estimates of the causes of death in children Lancet 2005 365 1147 1152 10.1016/S0140-6736(05)71877-8 15794969

[B9] Stein CE Birmingham M Kurian M Duclos P Strebel P The global burden of measles in the year 2000 - a model that uses country-specific indicators J Infect Dis 2003 187 suppl 1 S8 14 10.1086/368114 12721886

[B10] World Health Organization (WHO) Progress in reducing global measles deaths: 1999-2003 Wkly Epidemiol Rec 2005 80 78 81 15779712

[B11] Hersh B Beyond next steps in measles control5th Annual Measles Partners for Measles Advocacy Meeting; Geneva, Switzerland 2005

[B12] Schimmer B Ihekweazu C Polio eradication and measles immunisation in Nigeria Lancet Infect Dis 2006 6 63 65 10.1016/S1473-3099(06)70358-9 16439320

[B13] Omer SB Salmon DA Orenstein WA deHart P Halsey N Vaccine Refusal, Mandatory Immunization, and the Risks of Vaccine-Preventable Diseases N Engl J Med 2009 360 1981 8 10.1056/NEJMsa0806477 19420367

[B14] Nyarko P Pence B Debpuur C Immunization status and child survival in rural Ghana Population Research Division Working Paper No. 147, Population Council, New York 2001

[B15] Omer SB Salmon DA Orenstein WA deHart P Halsey N Vaccine refusal, mandatory immunization and the risks of vaccine-preventable diseases N Engl J Med 2009 360 1981 1988 10.1056/NEJMsa0806477 19420367

[B16] WHO, UNICEF Global immunization vision and strategy, 2006-2015 2005 Geneva, Switzerland and New York, USA: WHO and UNICEF

[B17] WHO Report of the eight meeting of the Technical Consultative Group (TCG) on the Global Eradication of Poliomyelitis, Geneva, 24-25 April 2003 2003 Geneva, Switzerland: WHO

[B18] Brenzel L Wolfson LJ Fox-Rushby J Miller M Halsey NA Jamison DT, Breman JG, Measham AR, Alleyne G, Claeson M, Evans DB, Jha P, Mills A, Musgrove P Vaccine-preventable diseases Disease control priorities in developing countries 2006 Oxford University Press and The World Bank. Washington: WHO 21250343

[B19] United Nations Children's Fund (UNICEF) The State of the World's Children 2001 New York: UNICEF

[B20] National Population Commission (NPC) Nigeria Demographic and Health Survey 2003 2004 Calverton, Maryland: National Population Commission and ORC Macro

[B21] Diez-Roux AV Investigating neighbourhood and area effect on health Am J Publ Health 2001 91 783 789 10.2105/AJPH.91.11.1783 PMC1446876 11684601

[B22] Pearl M Braveman P Abrams B The relationship of neighbourhood socioeconomic characteristics to birthweight among five ethnic groups in California Am J Publ Health 2001 91 1808 1824 10.2105/AJPH.91.11.1808 PMC1446884 11684609

[B23] Case A Lubotsky D Paxson C Economic status and health in childhood: The origins of the gradient American Economic Review 2002 92 1308 1334 10.1257/000282802762024520 29058397

[B24] Currie J Stabile M Socioeconomic status and child health: Why is the relationship stronger for older children? American Economic Review 2003 93 1813 1823 10.1257/000282803322655563 29058847

[B25] S-H Lee Demand for immunization, parental selection, and child survival: Evidence from rural India Review of Economics of the Household 2005 3 171 197 10.1007/s11150-005-0709-x

[B26] Sugathan KS Mishra V Retherford RD Promoting institutional deliveries in rural India: The role of antenatal-care services National Family Health Survey Subject Reports, No. 20 2001 Mumbai: Institutional Institute for Population Sciences; and Honolulu: East-West Center

[B27] StataCorporation Stata Statistical Software 2001 College Station, TX

[B28] Snijders T Bosker RJ Multilevel analysis - an introduction to basic and advanced multilevel modelling 1999 Thousand Oaks, California: Sage Publications

[B29] Larsen K Merlo J Appropriate assessment of neighborhood effects on individual health: integrating random and fixed effects in multilevel logistic regression Am J Epidemiol 2005 161 81 88 10.1093/aje/kwi017 15615918

[B30] Center for Multilevel Modelling MLwiN software package Centre for Multilevel Modelling 2000

[B31] Rashbash J Browne W Goldstein H Yang M Plewis I Healy M Woodhouse G Draper D Langford I Lewis T A users guide to MLwiN. Version 2.1 2000 London, Institute of Education

[B32] Chukwuezi B Through thick and thin: Igbo rural-urban circularity, identity and investment J Contemp Afr Stud 2001 19 55 55 10.1080/02589000124437

[B33] Nigerian Institute of Social and Economic Research (NISER) Nigerian Migration and Urbanization Survey 1993 Ibadan 1993

[B34] Antai D Faith and Child Survival: The Role of Religion on Childhood Immunization in Nigeria J biosocial Sci 2009 41 57 76 10.1017/S0021932008002861 18471339

[B35] Njoku G Measles immunization campaign targets 29 million Nigerian children At a glance: Nigeria. UNICEF 2006 http://www.unicef.org/infobycountry/nigeria_36211.html

[B36] Lynch JW Kaplan GA Cohen RD Kauhanen J Wilson TW Smith NL Salonen JT Do cardiovascular risk factors explain the relationship between socioeconomic status, risk of all-cause mortality, cardiovascular mortality and acute myocardial infarction? Am J Epidemiol 1996 144 934 942 8916504 10.1093/oxfordjournals.aje.a008863

[B37] Khan KS Wojdyla D Say L Gulmezoglu AM Van Look PF WHO analysis of causes of maternal death: a systematic review Lancet 2006 36 1066 1074 10.1016/S0140-6736(06)68397-9 16581405

[B38] World Health Organization (WHO) Maternal mortality in 2000; estimates developed by WHO, UNICEF and UNFPA 2003 Geneva: World Health Organization

[B39] Filmer D Pritchett LH Estimating wealth effects without expenditure data-or tears: an application to educational enrolments in states of India Demography 2001 38 115 132 11227840 10.1353/dem.2001.0003

